# Shikonin reactivates TSGs *GADD45B* and *PPP3CC* to block NSCLC cell proliferation and migration through JNK/P38/MAPK signaling pathways

**DOI:** 10.1186/s12906-023-04306-z

**Published:** 2024-01-02

**Authors:** Yujia Zhao, Dan Wu, Zhenkai Fu, Wenna Liu, Yu Yao, Ying Liang

**Affiliations:** 1 Department of Medical Oncology, the First Affiliated Hospital of Xi’an Jiaotong University, 710086 Xi’an, Shaanxi China; 2 Precision Pharmacy & Drug Development Center, Department of Pharmacy, Tangdu Hospital, Fourth Military Medical University, 710038 Xi’an, Shaanxi China; 3 School of Basic Medical Sciences, Peking University, 100191 Beijing, China

## Abstract

**Background:**

Shikonin, a natural naphthoquinone compound extracted from the Chinese traditional herbal medicine “Lithospermum erythrorhizon”, possesses antitumor activity against various cancer types. Tumor-suppressor genes (TSGs) negatively regulate cell growth, proliferation, and differentiation, thereby inhibiting tumor formation. However, the molecular mechanism of action of shikonin on TSGs in non–small-cell lung cancer (NSCLC) remains unclear.

**Methods:**

The inhibitory effect of shikonin on the proliferation and migration abilities of lung cancer cells were measured by Cell Counting Kit 8 (CCK8) and wound healing assays. The alteration of genes by shikonin treatment was detected by mRNA high-throughput sequencing and further confirmed by qPCR and western blotting experiments. The dominant functions of the upregulated genes were analyzed by GO and KEGG profiling.

**Results:**

Shikonin inhibited the proliferation and migration of A549 and H1299 NSCLC cells in a dose-dependent manner. mRNA high-throughput sequencing revealed a total of 1794 upregulated genes in shikonin-treated NSCLC cells. Moreover, bioinformatic analysis of GO and KEGG profiling revealed that the up-regulated genes were mostly involved in the JNK/P38/MAPK signaling pathway, among which the expression of GADD45B and PPP3CC was significantly enhanced. Finally, we confirmed that *GADD45B* and *PPP3CC* were indeed upregulated in JNK/P38/MAPK pathway.

**Conclusions:**

Taken together, these results suggested that shikonin might affect the expression of *GADD45B* and *PPP3CC* through the JNK/P38/MAPK pathway, therefore exerting an inhibitory effect on the proliferation and migration of cancer cells. To our knowledge, this is the first study reporting the role of shikonin in upregulating TSGs to activate the JNK/P38/MAPK signaling pathways in NSCLC.

**Supplementary Information:**

The online version contains supplementary material available at 10.1186/s12906-023-04306-z.

## Introduction

Lung cancer is one of the most diagnosed cancers, with the highest mortality rate among malignancies worldwide. Globally, approximately 2 million new cases and more than 1.76 million deaths are recorded each year [1, 2]. Traditionally, lung cancer is histologically classified into small-cell lung cancer and non–small-cell lung cancer (NSCLC), with NSCLC consisting predominantly of lung adenocarcinoma (LUAD) and lung squamous cell carcinoma. NSCLC is the main histological subtype accounting for 85% of lung cancers [3]. According to statistics, approximately two-thirds of patients with NSCLC are in the middle and late stages at the time of diagnosis, losing the best time for treatment. Current clinical treatments for NSCLC mainly contain surgery, chemotherapy, radiation therapy, targeted therapy, and immunotherapy; however, these treatments still do not significantly prolong patient survival and may cause side effects such as liver and kidney damage, decreased resistance to infection, and anemia [3–5], making it critical to find safer and more effective treatment strategies. As current treatment strategies for NSCLC face issues, such as drug resistance and body tolerance, it is vital to identify additional treatment strategies for the prevention and treatment of NSCLC. Traditional Chinese medicine (TCM) is characterized by its novel pharmacological mechanisms, low toxicity, and limited side-effects. An increasing number of researchers have recently focused their efforts on discovering active TCM-derived anticancer ingredients [6–8].

Shikonin, often referred to as “Zicao” in TCM, is the major bioactive ingredient extracted from the roots of *Lithospermum erythrorhizon*. The chemical structure of shikonin is 5,8-dihydroxy-2-((1R)-1-hydroxy-4-methy1-3-penteny1)-1,4-naphthoquinone [9]. Shikonin has been reported to have various pharmacologic roles, such as antifungal, antibacterial, antitumor, and wound-healing properties [10–12]. Recently, a myriad of studies has documented that shikonin exhibits solid antitumor functions against various types of cancer, such as lung, breast, gastrointestinal and urogenital cancers. The underlying mechanisms mostly involve suppression of cell proliferation, induction of apoptosis, and blockade of cell migration and invasion through various molecular pathways [9]. For instance, Wang et al. found that shikonin suppress the growth of H1299 lung adenocarcinoma cells through targeting survivin and modulating the expression of apoptosis-related and CDK/cyclin family members, inducing apoptotic cell death and arresting the cell cycle [10]. Zang et al. reported that shikonin can induce cell apoptosis to inhibit the growth of paclitaxel-resistant NSCLC through blocking NEAT1 and Akt signaling [13]. Given the important role of shikonin in lung cancer treatment, it is particularly important to further explore its mechanism of action.

Tumor-suppressor genes (TSGs) can inhibit the occurrence and development of tumors by participating in DNA damage repair, inhibiting cell division, inducing apoptosis, and inhibiting metastasis [14, 15]. Inactivation of TSGs is a common mechanism leading to the occurrence of cancer. Abnormal expression or inactivation of TSGs, because of mutations or other reasons, abolishes their inhibitory effect, resulting in unlimited cell proliferation and leading to tumorigenesis [15]. According to the literature, TSGs extensively participate in inhibiting the development of lung cancer [16]. Foster et al. demonstrated that ATMIN acts as a tumor suppressor in LUAD, and deletion of *Atmin* increasing tumor burden in a LUAD mouse model [17]. Han et al. confirmed that as a tumor suppressor, MIR99AHG interacts with *ANXA2* to accelerate the generation of AnxA2-induced ATG16L + vesicles, thereby promoting the assembly of phagocytes and inhibiting the proliferation and metastasis of LUAD cells in vitro and in vivo [18]. Wang et al. demonstrated that *SH3GL3* acts as a TSG by inhibiting the cell cycle at the G0/G1 phase and inducing apoptosis in lung cancer cells; as such, it was indicated as a biomarker for the diagnosis and prognosis of lung cancer [19]. In view of the important role of TSGs in lung cancer, it is not clear whether shikonin further affects the development of lung cancer by reactivating TSGs. Therefore, we attempted to elucidate the mechanism of action of shikonin in the inhibition of lung cancer from a novel perspective, with a view to finding a new target for the treatment of NSCLC in the clinic as well as providing theoretical justifications for the mechanism of inhibition of lung cancer by comfreyin.

In the present study, we confirmed that shikonin suppressed the proliferation and migration of A549 and H1299 LUAD cells in a dose-dependent manner. Then, we creatively crossed the high-throughput sequencing data of the lung cancer cells treated with shikonin with the TSGs that are lowly expressed in lung cancer in the TCGA database, and found that the inhibitory effect of shikonin on NSCLC might be related to TSGs. KEGG analysis showed that shikonin-regulated TSGs mainly inhibited lung cancer cells through the MAPK signaling pathway. Furthermore, qPCR and western blot experiments confirmed that TSGS, such as *DUSP5*, *GADD45B*, and *PPP3CC*, were upregulated in shikonin-treated lung cancer cells. Finally, we further validated the expression of *GADD45B* and *PPP3CC* in NSCLC using the TCGA database and KM survival curve analysis, and found that upregulated *GADD45B* and *PPP3CC* improved prognosis. Therefore, we hypothesized that the suppressive effects of shikonin on the proliferation and migration of A549 and H1299 cells are mediated through its interactions with *GADD45B* and *PPP3CC*, concurrently activating the MAPK pathway. Our study suggested that shikonin is an attractive candidate for further consideration in the development of anti-lung cancer drugs.

## Materials and methods

### Reagents and antibodies

Shikonin (MB7082; purity > 98%; Meilunbio, Dalian, China) was dissolved in DMSO to prepare a 1000 µM stock solution that was stored at − 20 °C. The concentration of DMSO in the solution did not exceed 0.2%. The RPMI-1640 cell culture medium and RPMI-1640 complete medium (1% HEPES 1 M buffer solution, 1% sodium pyruvate, and 1% glutamine) were purchased from CYTIVA (SH30809.01). Antibodies against GADD45B (DF2375, Affinity Biosciences, Jiangsu, China), PPP3CC (19653-1-AP, Proteintech, Wuhan, China) and β-actin (WH256886, ABclonal, Wuhan, China) were purchased from Affinity Biosciences (DF2375), Proteintech (19653-1-AP) and ABclonal (WH256886), respectively, whereas antibodies against p38 MAPK (CST9212S, Cell Signaling Technology, Boston, USA), JNK (CST9252S, Cell Signaling Technology, Boston, USA) and ERK1/2 (CST4695S, Cell Signaling Technology, Boston, USA) were all purchased from Cell Signaling Technology.

### Cell culture

The A549 and NCI-H1299 human lung adenocarcinoma cell lines were purchased from the Cell Bank of the Chinese Academy of Science (Shanghai, China). Cells were cultured in RPMI-1640 and RPMI-1640 complete medium, respectively, both of which were supplemented with 10% fetal bovine serum (FBS, Gibico, 10099-141, Australia) and 1% penicillin–streptomycin in a humidified atmosphere of 5% CO_2_ at 37 °C.

### Cell counting kit 8 (CCK8) assay

Cell viability was assessed using the CCK8 assay (Cell Counting Kit 8, HY-K0301, MedChemExpress). Cells were cultured overnight in 96-well plates at 1 × 10^4^ cells/well in a volume of 100 µl and then treated with various concentrations of shikonin with 0, 0.3, 0.9, 2.7 and 8.1 µM for 24 h, respectively. Subsequently, 10 µl CCK8 was added to each well and incubated for 20 m at 37 ℃. The absorbance of each well at 450 nm was recorded by enzyme-labeled instrument, and cell viability was calculated using wells without cells as blank. IC50 values were obtained from nonlinear regression curve fit analysis by GraphPad Prism 5.0 (Version7.0, GraphPad Software, Inc).

### Colony-formation assay

Cells were diluted in culture medium and seeded in 6-well plates at a density of 3 × 10^3^ cells per well. After incubation for 24 h, A549 and H1299 cells were treated with either 3 µM shikonin, 6 µM shikonin, respectively or an equal volume of culture medium as a control for 15 days. Grown colonies were stained with crystal violet and photographed. The colony area was calculated using the ImageJ software (Version1.52a, National Institutes of Health).

### Wound healing assay

A549 and H1299 cells were cultured at 1 × 10^4^/well in the 6-well plates until they reached the confluence of 90–95%. Then a vertical wound was made with sterile 1 ml pipet tip. The floating cells were washed with PBS, and medium with or without 3 µM and 6 µM shikonin was added to the remaining A549 and H1299 cells, respectively. The images of wound in each well were taken by microscope at 0 h, 24 and 48 h. Areas of the wound were measured by Image J and the data are expressed as the ratio of the healed wound area at 24 and 48 h compared to initial wound area (0 h).

### Reverse transcription polymerase chain reaction (RT-PCR) detection

A549 and H1299 cells were treated with 3 and 6 µM shikonin for 24 h, respectively. Total RNA was isolated using TRIzol (B511311-0100; Sangon Biotech) and reverse transcribed into cDNA with oligo (dT18) primers using the PrimeScript RT Reagent Kit with gDNA Eraser (Takara; Japan) under the manufacturer’s instructions. The relative expression of target genes was determined after normalization to the expression of the internal control *GAPDH* using the 2^-ΔΔCt^ method. The sequences of primers used in RT-PCR are as follows: *DUSP5* forward primer, 5’-TCACCTCGCTACTCGCTTGC-3’ and reverse primer, 5’-GATGAGGGCTCTCTCACTCT-3’; *PPP3CC* forward primer, 5’-GTACATGGAGGAATGTCAC-3’ and reverse primer, 5’-CTCATTGCCATAATCCTCTG-3’; *GADD45B* forward primer, 5’-GGCCGCTCAGCGCCAGGATC-3’ and reverse primer, 5’-GAGCGTGAAGTGGATTTGCA-3’; *DUSP9* forward primer, 5’- CAGCCGTTCTGTCACCGTC-3’ and reverse primer, 5’-CAAGCTGCGCTCAAAGTCC-3’; *GAPDH* forward primer, 5’-GCCTTCTCCATGGTGGTGA-3’ and reverse primer, 5’-ACCGTCAAGGCTGAGAAC-3’.

### Western blot analysis

A549 and H1299 Cells were cultured in the 6-well plates with various concentrations of shikonin at 3, 6 µM and 6, 12 µM, respectively until they reached confluence of 90–95%. Total proteins were extracted by using radioimmunoprecipitation assay (RIPA) buffer, and the concentration was determined using BCA Protein Assay Kit (Beyotime). Equal amounts of protein were separated by 4–12% gradient sodium dodecyl sulfate (SDS)-polyacrylamide gel and transferred to polyvinylidene difluoride (PVDF) membranes (Millipore). The membrane was blocked with 5% milk. The blot was then incubated with primary antibodies overnight at 4 ℃ and then incubated with anti-rabbit secondary antibodies (1/20,000 dilution) for 1 h on the next day. The immunoreactions were measured by an enhanced chemiluminescence (ECL) system with the manufacturer’s instructions. The blots are cut prior to hybridisation with antibodies during blotting, the images of all blots are provided in the Supplementary file.

### RNA preparation and mRNA high-throughput sequencing analysis

To identify the difference of gene expression in response to shikonin exposure, A549 lung cancer cells were cultured over night at 3 × 10^5^ cells/well in 6 cm dish and incubated at 37 °C. The cell samples were prepared in triplicate. They were then incubated with 3 µM shikonin or blank cell medium. After 24 h of incubation, the total RNA was extracted by TRIzol (B511311-0100; Sangon Biotech) to perform high-throughput sequencing. Then, the concentration and quality of purified RNA were detected by using Nanodrop 2000 (Thermo Scientific) and the Bioanalyzer 2100 (Agilent Technologies) for further library preparation. The different expression genes (DEGs) were performed with DESeq2 (version 3.12) in R (version 4.0). The detail profiling methods are referred in our previous study [20]. Fold change > 1.5, *P* < 0.05. The accession number for the sequencing data generated in this paper is GSE222640 and can be gained from GEO (Gene Expression Omnibus) datasets. The bioinformatic profiling data for DEG and functional enrichment are all presented in Supplemantary Information.

### Statistical analysis

All data are presented as the mean ± SEM, and each experiment was independently repeated 3 times unless otherwise noted. Statistical analyses were performed using GraphPad Prism 5.0 (Version7.0, GraphPad Software, Inc) by two-tailed Student t-test. **** represents *p* < 0.0001, *** represents *p* < 0.001, ** represents *p* < 0.01 and * represents *p* < 0.05. *p* < 0.05 were defined as significant.

## Results

### Shikonin blocked the proliferation and migration of lung adenocarcinoma cells

**Fig. 1:**
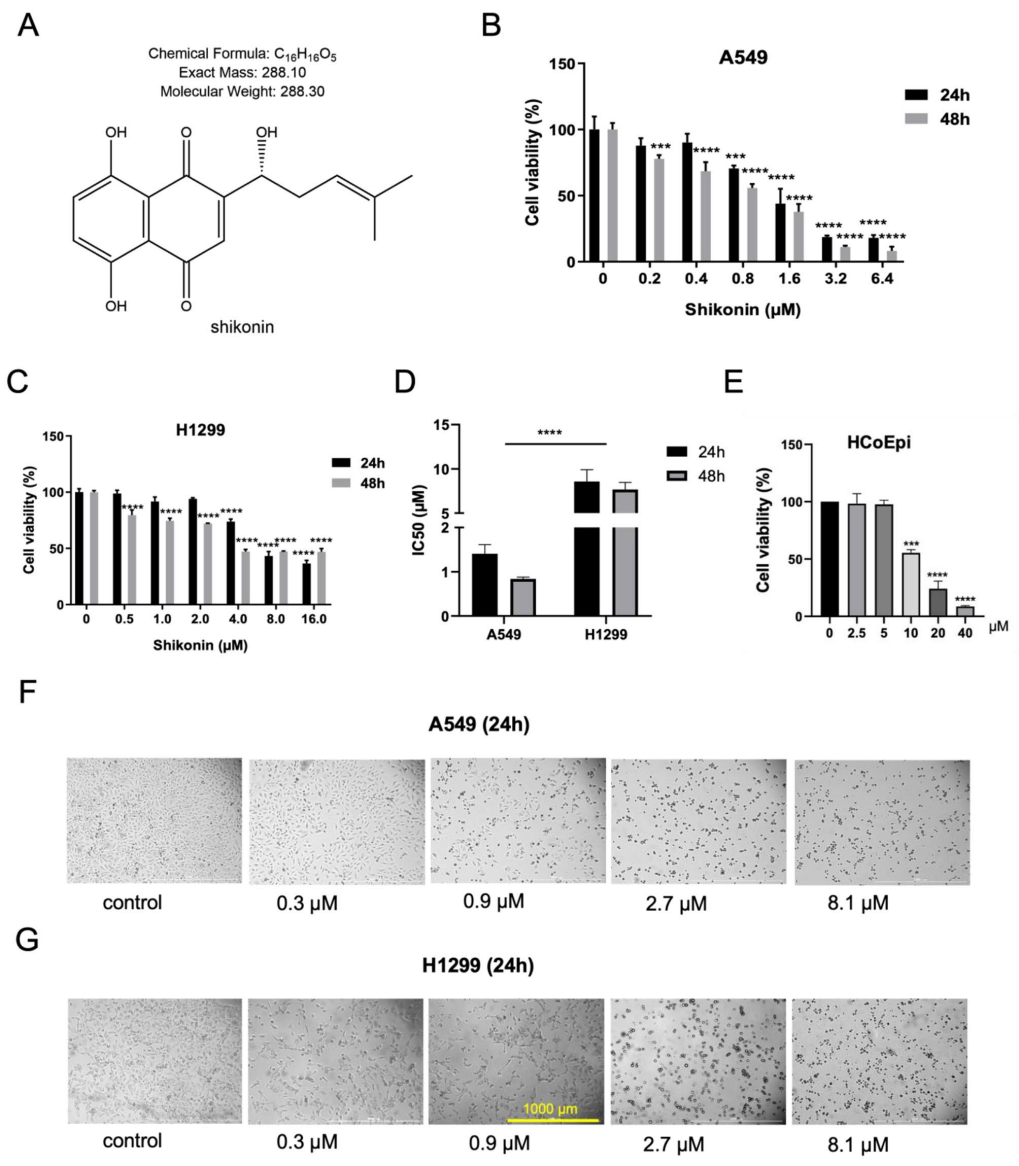
Shikonin inhibits the cell viability of A549 and H1299 cells in dose-dependent manner. **A.** The chemical structure of shikonin. **B-C.** The cell viability of A549 and H1299 cells was determined by CCK8 assays. **D.** The respective IC50 in A549 and H1299 cells was calculated. **E. **The cell viability of HCoEpi cells was determined by a CCK8 assay. **F-G.** Shikonin was able to inhibit the cell growth of A549 and H1299 cells in a dose-dependent manner. **** represents *p* < 0.0001, *** represents *p* < 0.001

The chemical structure of shikonin, a potential anticancer agent, is depicted in Fig. 1A. To assess the effect of shikonin on the viability of lung adenocarcinoma cells, we treated A549 and H1299 cells with serial dilutions of shikonin (0, 0.2, 0.4, 0.8, 1.6, 3.2, 6.4 µM and 0, 0.5, 1.0, 2.0, 4.0, 8.0, 16.0 µM, respectively) for 24 and 48 h. We determined cell viability using the CCK8 assay, and found that shikonin inhibited cell growth in a dose-dependent manner (Fig. 1B and C); in particular, we detected that the growth of cells declined in a stepwise manner with increasing doses of shikonin (Fig. 1F and G). The respective IC50 values in A549 and H1299 cells were 1.41 µM and 8.58 µM at 24 h, and 0.84 µM and 7.67 µM at 48 h (Fig. 1D). Interestingly, we noticed that the IC50 values of shikonin on A549 and H1299 cells between 24 and 48 h were not significantly different, indicating that the inhibitory effect of shikonin on A549 and H1299 cells was not time-dependent. In order to further distinguish the anti-proliferative activity of shikonin from its cytotoxic effects, we detected the IC50 value of shikonin on normal colon epithelial cells HCoEpic at 24 h as 11.35 µM (Fig. 1E), which is obviously larger than that of A549 and H1299 cells. This indicates the inhibitory function of shikonin on lung adenocarcinoma cells is not contributed by its cytotoxic effects.

**Fig. 2:**
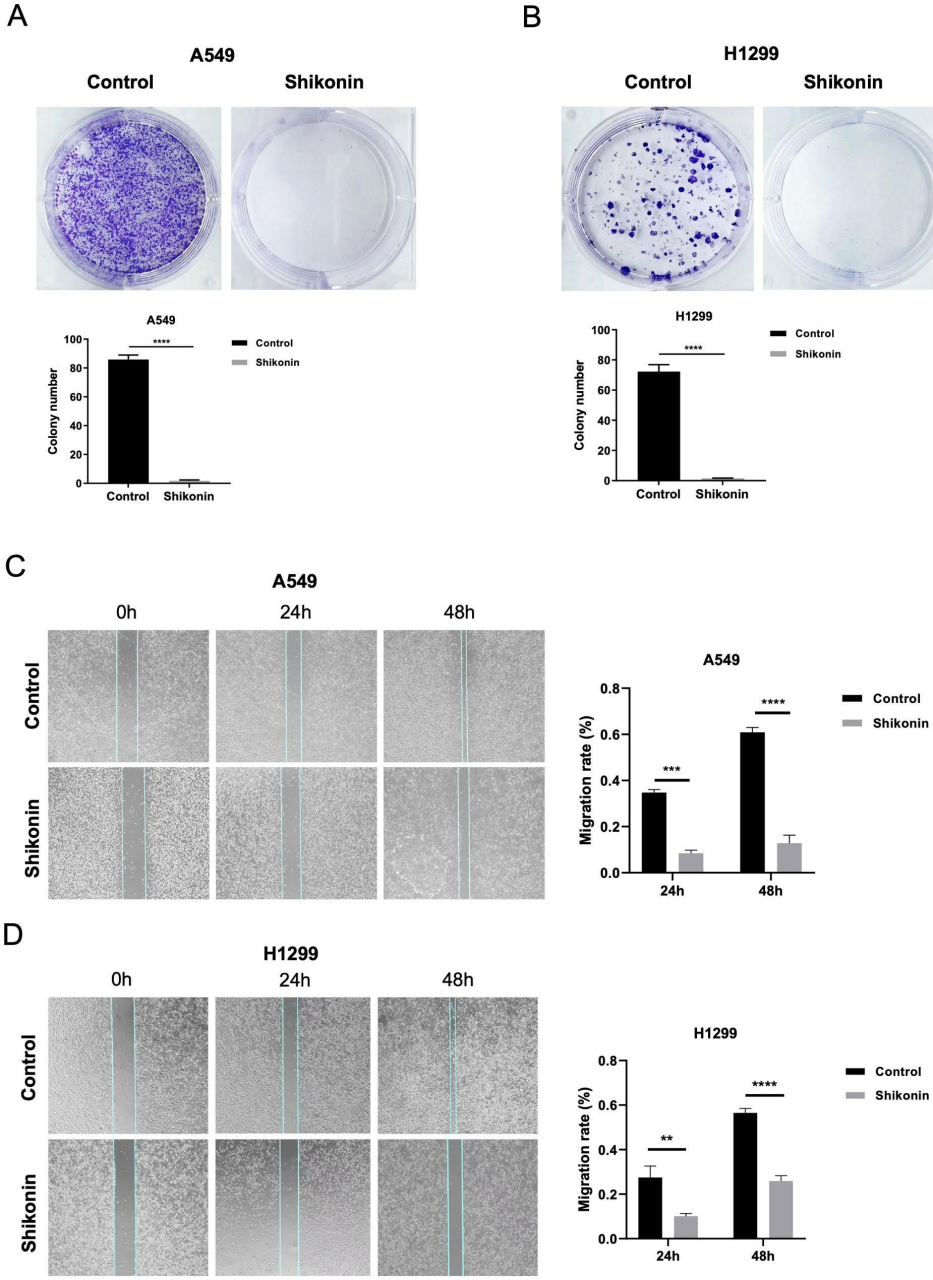
Shikonin inhibits the proliferation and migration abilities of A549 and H1299 cells. **A-B.** Shikonin significantly decreased the colony numbers of A549 and H1299 cells in colony formation assays. **C-D.** The migration viability of A549 and H1299 cells was significantly blocked by shikonin at 24 and 48 h. Images were gained under magnification x200. **** represents *p* < 0.0001, *** represents *p* < 0.001, ** represents *p* < 0.01

Furthermore, we determined how shikonin plays a role on cell proliferation by using colony formation and wound-healing assays. We treated A549 and H1299 cells with 3 and 6 µM shikonin, respectively. As shown in Fig. 2A and B, both A549 and H1299 cells showed high sensitivity to shikonin, as the number and size of colonies were significantly decreased compared with those in the control group (upper panel of Fig. 2A and B). We found that the colony numbers of A549 cells in the control and shikonin treatment groups were 343 and 6, respectively, while the colony numbers of H1299 cells were 72 and 1, respectively (lower panel of Fig. 2A and B). Moreover, we performed a wound healing assay to investigate whether shikonin inhibits cell migration by treating A549 and H1299 cells with 3 and 6 µM shikonin, respectively. We accordingly found that the migration ability of cells was obviously inhibited after shikonin treatment at 24 and 48 h, as indicated in the left panels of Fig. 2C and D. Compared with the control group, we observed a significant reduction in the number of migrated cells in the treated groups at 24 and 48 h (right panels of Fig. 2C and D).

### Shikonin modulated the expression of tumor-suppressor genes through MAPK signaling by high-throughput sequencing

**Fig. 3:**
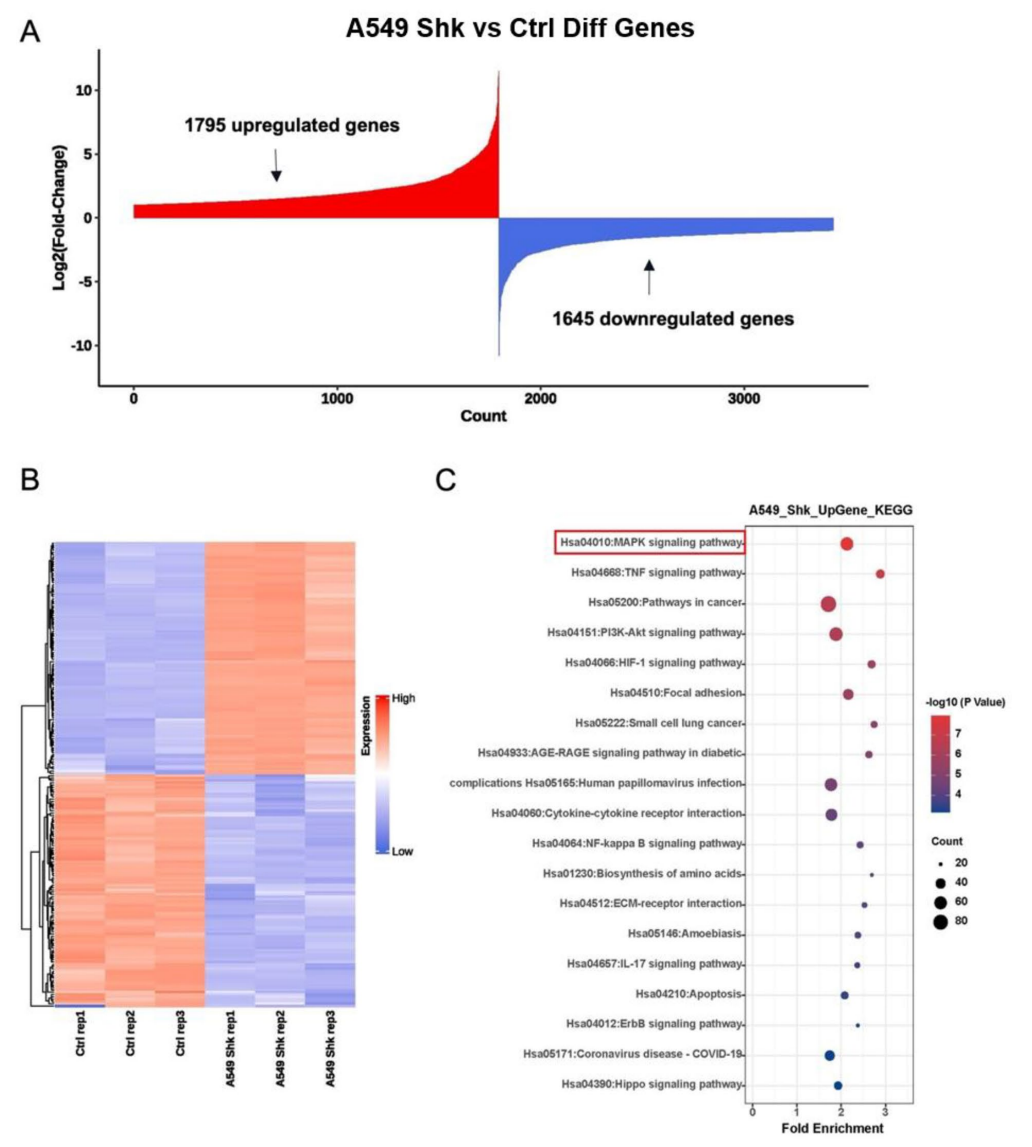
Shikonin is related to TSG expression alterations through MAPK signaling. **(A)** A total of 1795 upregulated genes and by 1645 downregulated genes by Shikonin in A549 cells through mRNA sequencing analysis. **(B)** Heatmap of the top 100 changed genes by shikonin. **(C)** The upregulated genes are mainly involved in MAPK signaling pathway by KEGG profiling

To investigate the fundamental molecular mechanism by which shikonin suppresses the proliferation and migration abilities of lung adenocarcinoma cells, we performed mRNA high-throughput sequencing profiling of A549 cells. As shown in Fig. 3A, a total of 1795 genes were upregulated by shikonin, whereas 1645 genes were downregulated. The top 100 upregulated and downregulated genes are depicted in Fig. 3B. The main molecular function of the top upregulated genes in KEGG profiling was the MAPK signaling pathway shown in Fig. 3C, which was consistent with the findings of previous studies on the shikonin-induced activation of the MAPK pathway [21, 22].

**Fig. 4:**
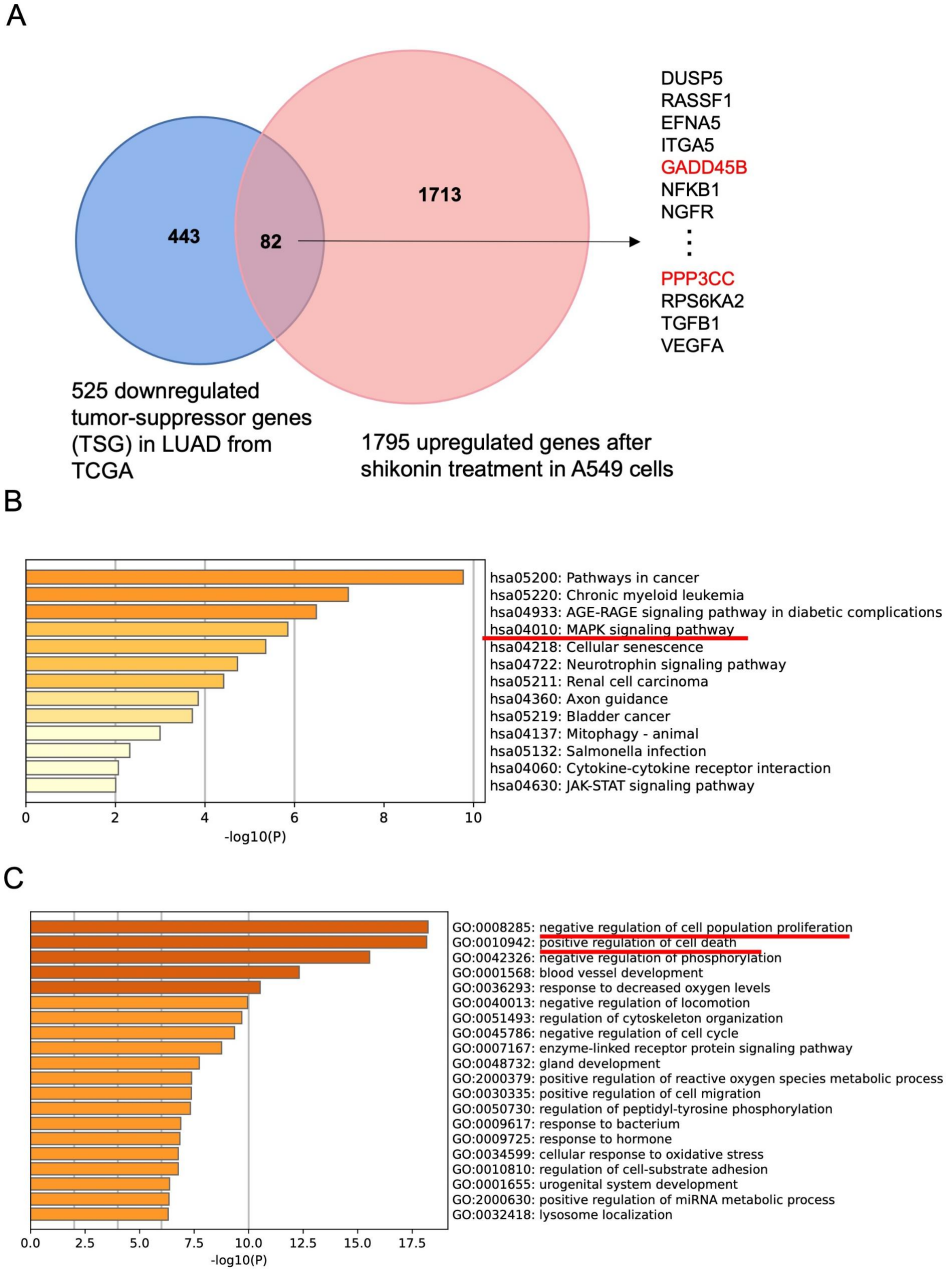
Shikonin may reactivate TSGs in LUAD through MAPK signaling. **(A)** A total of 82 activated TSGs among 1795 upregulated genes by shikonin. **(B)** KEGG profiling for the 82 upregulated TSGs by shikonin. **(C)** GO analysis for the 82 upregulated TSGs by shikonin

Tumor-suppressor genes are frequently downregulated in various types of cancers. Loss of function of TSGs contributes to carcinogenesis; conversely, rescue of downregulated TSGs might block the progression of cancer cells [14]. To investigate whether shikonin activates TSGs in lung adenocarcinoma, we explored the expression of TSGs in lung adenocarcinoma samples from the TSGene database (https://bioinfo.uth.edu/TSGene/) and identified that a total of 525 TSGs were downregulated [23, 24]. Interestingly, we intersected 1,795 differential genes that were upregulated after shikonin treatment with these 525 TSGs that were lowly expressed in lung adenocarcinoma samples, and a total of 82 TSGs were obtained, including *DUSP5*, *PPP3CC*, and *GADD45B*, among the 1795 upregulated genes after shikonin treatment, indicating that these 82 TSGs that were downregulated in lung adenocarcinoma were reactivated by shikonin (Fig. 4A). KEGG pathway analysis in Fig. 4B demonstrated that the 82 activated TSGs are mainly involved in cancer and MAPK pathways, which is consistent with the top 100 upregulated genes in Fig. 3C. Moreover, Gene Ontology (GO) profiling showed that these 82 upregulated TSGs are typically related to negatively regulating cell proliferation or positively leading to cell death (Fig. 4C), suggesting that their activation suppresses cell growth and proliferation.

### Expression of Tumor suppressors *PPP3CC* and *GADD45B* in MAPK signaling was significantly increased by shikonin treatment

**Fig. 5:**
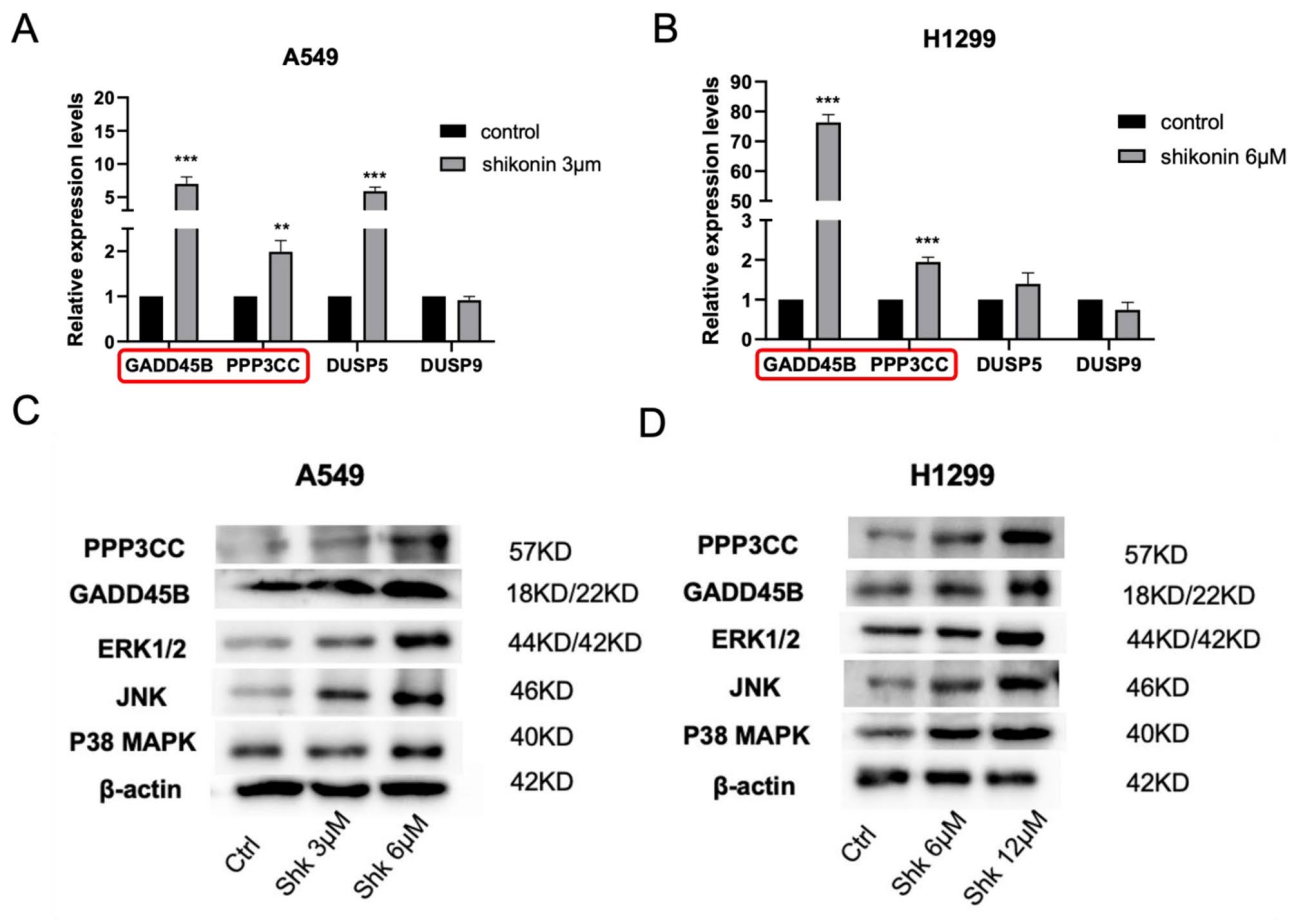
Shikonin increased tumor suppressor genes *PPP3CC* and *GADD45B * in MAPK signaling. **A-B.** *GADD45B* and *PPP3CC* are activated after shikonin treatment in both A549 and H1299 cells by qPCR detection. **C-D.** The MAPK proteins, GADD45B and PPP3CC are activated after shikonin treatment in both A549 and H1299 cells by western blotting. The blots are cut prior to hybridisation with antibodies during blotting and the cropped plots are used in the figure to improve the clarity of presentation. *** represents *p* < 0.001, ** represents *p* < 0.01

To further verify the activation of TSGs by shikonin, we randomly selected 4 TSGs, namely *DUSP5*, *DUSP9*, *GADD45B* and *PPP3CC*, from the 82 upregulated TSGs and detected their expression using RT-PCR. We found that shikonin treatment led to the activation of *GADD45B*, and *PPP3CC* in both A549 and H1299 cells, whereas the expression of *DUSP9* was not significantly changed and *DUSP5* was only upregulated in A549 cells (Fig. 5A and B). Interestingly, among these selected TSGs, *GADD45B* and *PPP3CC* have been reported as tumor suppressors that inhibit cancer cell proliferation and invasion in various cancers; however, their roles in lung adenocarcinoma remain unknown. Therefore, we preferentially selected *GADD45B* and *PPP3CC* as candidate targets for shikonin in lung adenocarcinoma cells. We also performed western blotting to verify the levels of protein expression of *GADD45B* and *PPP3CC* after shikonin treatment. As expected, we detected that the expression of *GADD45B* and *PPP3CC* was obviously increased when A549 and H1299 cells were treated with 3, 6 µM and 6, 12 µM shikonin, respectively. Moreover, we observed the activation of marker proteins, such as ERK1/2, JNK, and P38 MAPK, after shikonin treatment, indicating that the MAPK signaling pathway was indeed positively modulated by shikonin.

**Fig. 6:**
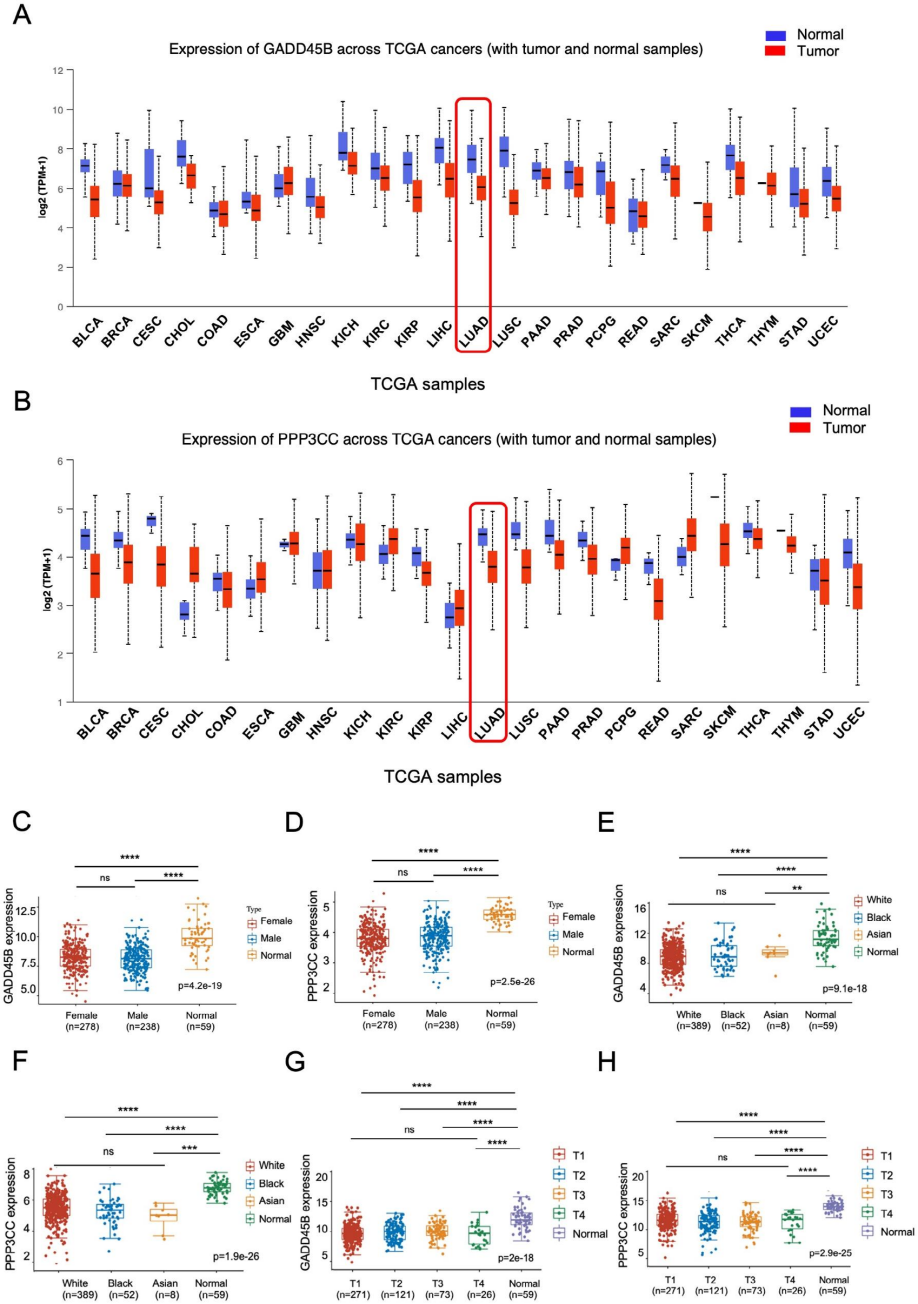
Expression of tumor suppressor genes *PPP3CC* and *GADD45 B* in pan-cancer. **A-B.** Pan cancer gene expression profile of *GADD45B* and *PPP3CC.* Red boxplot depicts expression level in primary tumors, while blue boxplot indicate expression in normal samples. **C-H.** The expression distribution of *GADD45B* and *PPP3CC* in tumor and normal tissues from TCGA database. The downregulation of *GADD45B* and *PPP3CC* are not related to gender, race and tumor stages. The statistical differences of two and three groups were tested through the Wilcox test and Kruskal-Wallis test, respectively. **** represents *p* < 0.0001, *** represents *p* < 0.001, ** represents *p* < 0.01

To determine whether the expression of *GADD45B* and *PPP3CC* was downregulated across various TCGA cancers, we investigated the pancancer gene-expression profiles of *GADD45B* and *PPP3CC* using the UALCAN database (http://ualcan.path.uab.edu/) [25]. As shown in Fig. 6A and B, the expression of both *GADD45B* and *PPP3CC* was remarkably downregulated in lung adenocarcinoma, as well as in numerous other cancer types, in line with the reported downregulation of *GADD45B* and *PPP3CC* in various cancers [26, 27]. Moreover, we wonder whether their expression distributions are correlated with gender, race and tumor stages or not, so we interrogated the RNA-seq expression data of lung adenocarcinoma and the corresponding clinical information from TCGA datasets (https://portal.gdc.com). As seen in Fig. 6C and D, the expression of both *GADD45B* and *PPP3CC* was not related to gender, that is, no matter what gender is, their expressions were distinctly lower in lung adenocarcinoma than those of normal ones. The results are the same for races and tumor stages as presented in Fig. 6E-F and G-H, respectively.

Taken together, all these results demonstrated that both *GADD45B* and *PPP3CC* were downregulated in lung adenocarcinoma as TSGs and could be activated by shikonin through the MAPK signaling pathway.

### *PPP3CC* and *GADD45B* were correlated with patient prognosis

**Fig. 7:**
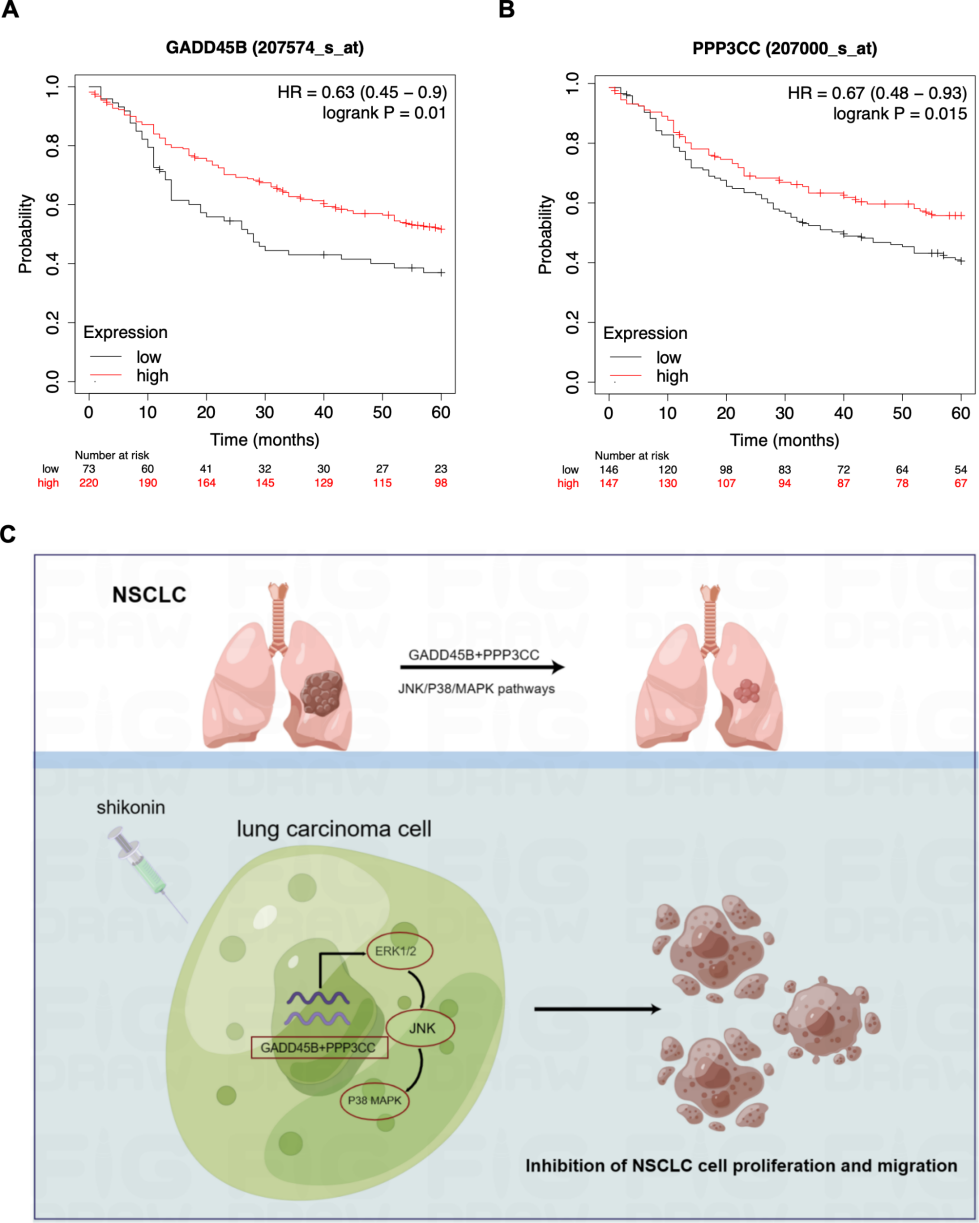
TSGs *GADD45 B* and *PPP3CC * are correlated with patient prognosis in clinic. **A-B.** *GADD45B* and *PPP3CC* expression was positively correlated with patient survival outcomes by Kaplan–Meier plots. **C.** The schematic diagram of the present study. Shikonin treatment activates the expression of *GADD45B* and *PPP3CC* through the JNK/P38/MAPK pathway, thus inhibiting the growth and migration of NSCLC cells

As *GADD45B* and *PPP3CC* are activated by shikonin to suppress cell proliferation and metastasis in lung adenocarcinoma, we speculated whether their expression was correlated with patient prognosis. Hence, we analyzed Kaplan–Meier (KM) plots of overall survival to confirm the effects of the expression of *GADD45B* and *PPP3CC* on patient survival outcomes. As shown in Fig. 7A and B, patients with LUAD with higher levels of expression of *GADD45B* and *PPP3CC* exhibited better survival outcomes, suggesting that *GADD45B* and *PPP3CC* indeed exhibit a tumor-suppressive effect. Generally, we determined that the high expression of *GADD45B* and *PPP3CC* was correlated with better patient prognosis in KM analysis. Furthermore, we depicted the schematic diagram of the current research as shown in Fig. 7C. Shikonin activates the expression of *GADD45B* and *PPP3CC* through the JNK/P38/MAPK pathway to suppress the growth and migration abilities of NSCLC cells, providing an alternative mechanism for the inhibitory function of shikonin on NSCLC.

## Discussion

To investigate their potential for antitumor drug development, we need to understand the underlying molecular mechanism by which natural products modulate the proliferation and metastasis of cancer cells. Shikonin is an active naphthoquinone derived from the Chinese traditional medicine Zi Cao (*purple gromwell*) with antitumor, anti-inflammation, and antibacterial functions [28]. A Recent study has reported that shikonin suppresses cancer cell proliferation via the MAPK signaling pathway, which mediates the induction of apoptosis [21]. However, the detailed molecular mechanisms by which shikonin blocks the proliferation of lung cancer cells through the MAPK pathway are yet to be elucidated. In the current study, we focused on the molecular mechanisms underlying the suppressive effects of shikonin in human lung adenocarcinoma cell lines. Specifically, we found the upregulation of 1795 genes in A549 cells by shikonin, among which 82 TSGs were mainly correlated with the MAPK pathway, indicating a remarkable role of TSGs in NSCLC via the MAPK signaling pathway.

Importantly, TSGs encode critical intracellular regulators that control cell proliferation, cell growth, and DNA damage in cancer [29]. The first TSG *Rb1* (retinoblastoma gene 1), which encodes the retinoblastoma protein that causes RB cancer development, was discovered by Knudson, influences cell survival and apoptosis through interactions with other nuclear proteins [30]. Several studies have reported that *TP53* acts within the nucleus to regulate repair mechanisms and apoptosis following DNA damage; *VHL* plays a role in regulating the hypoxia-inducible factor complex, maintaining normal levels of blood vessel formation; *PTEN*’s phosphatase activity indirectly inhibits signaling through PI3K by dephosphorylating PIP3 back to PIP2 [31]. Although both shikonin and inactivation of TSGs are known to be closely related to cancer progression, whether TSGs can be modulated by shikonin remains unclear. In this study, high-throughput sequencing revealed that shikonin can activate TSGs in lung cancer cells through the MAPK pathway. Taking TSG *PPP3CC* and *GADD45B*, which are involved in the MAPK signaling pathway, as candidates, we found that the expression of both was upregulated by shikonin treatment, as indicated by qPCR and western blotting assays (Fig. 5). Moreover, KM analysis revealed that the expression of both *GADD45B* and *PPP3CC* was associated with the prognosis of patients with LUAD, consistent with findings from other studies that also reported their inhibitory functions on tumors [32–35]. For example, *GADD45B* was positively involved in G2/M-phase arrest and apoptosis through induction of cyclin-dependent kinase inhibitor 1 A expression [36]; and it has been reported to be a promising tumor suppressor in NSCLC and has close association with NSCLC development [34]. Meanwhile, downregulation of *PPP3CC* has been linked with an autophage-related prognostic signature for hepatocellular carcinoma, and suppression of *PPP3CC* by ZEB1 activates NF-kB signaling, leading to glioma cell proliferation and invasion [35], indicating that *PPP3CC* acts as a tumor suppressor in these cancers. Surprisingly, the present study is the first to demonstrate the suppressive role of *PPP3CC* in NSCLC cells. Hence, *PPP3CC* may serve as a novel therapeutic target for NSCLC.

In general, we have demonstrated that shikonin is capable of blocking the proliferation and metastasis of LUAD cells by activating TSGs, such as *PPP3CC* and *GADD45B*, through the MAPK signaling pathway (Fig. 7C). In particular, *PPP3CC* was a new target discovered in NSCLC. To the best of our knowledge, the present study is the first to find that shikonin can inhibit lung cancer progression by reactivating TSGs, providing novel insights into the molecular mechanism by which shikonin inhibits the proliferation of LUAD cells and paving the way for the development of patent medicines based on shikonin to be used in the clinical setting.

### Electronic supplementary material

Below is the link to the electronic supplementary material.

Supplementary Material 1

## Data Availability

The datasets supporting the conclusions of this article are included within the article. The original high throughput sequencing data generated in this article can be downloaded at: https://www.ncbi.nlm.nih.gov/geo/query/acc.cgi?acc=GSE222640 with accession number GSE222640 and token number szqrmawqnnsvhcf.
