# Supplementary material for: Shikonin reactivates TSGs GADD45B and PPP3CC to block NSCLC cell proliferation and migration through JNK/P38/MAPK signaling pathways
Source: BMC Complement Med Ther. 2024 Jan 2;24:10. doi: 10.1186/s12906-023-04306-z (PMC10759768; doi:10.1186/s12906-023-04306-z)
Supplement: Supplementary file 1 — Supplementary Material 1 [file 12906_2023_4306_MOESM1_ESM.pdf]

H1299 Cells

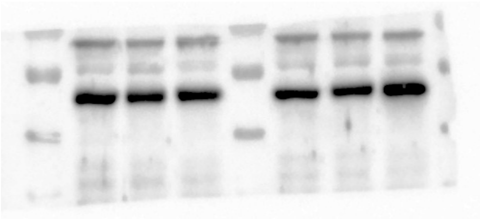

PPP3CC

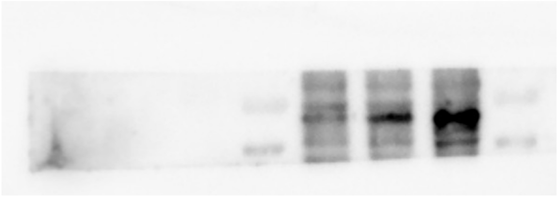

PPP3CC

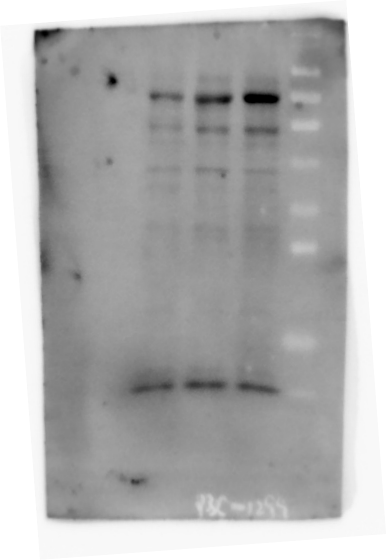

PPP3CC

H1299 Cells

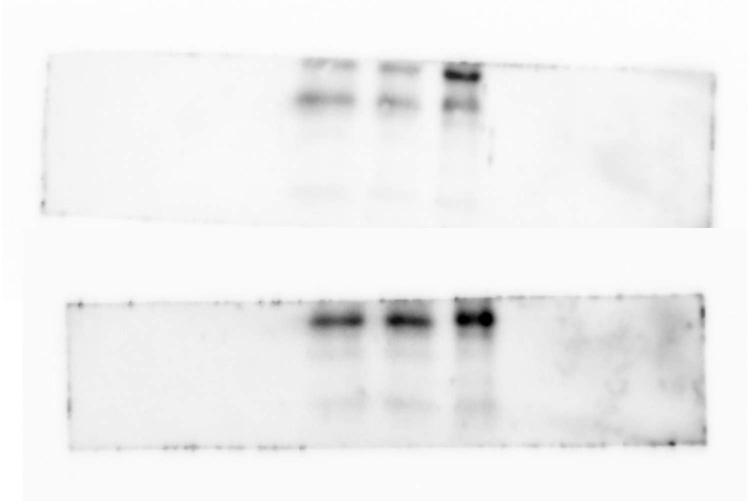

GADD45B

GADD45B

GADD45B

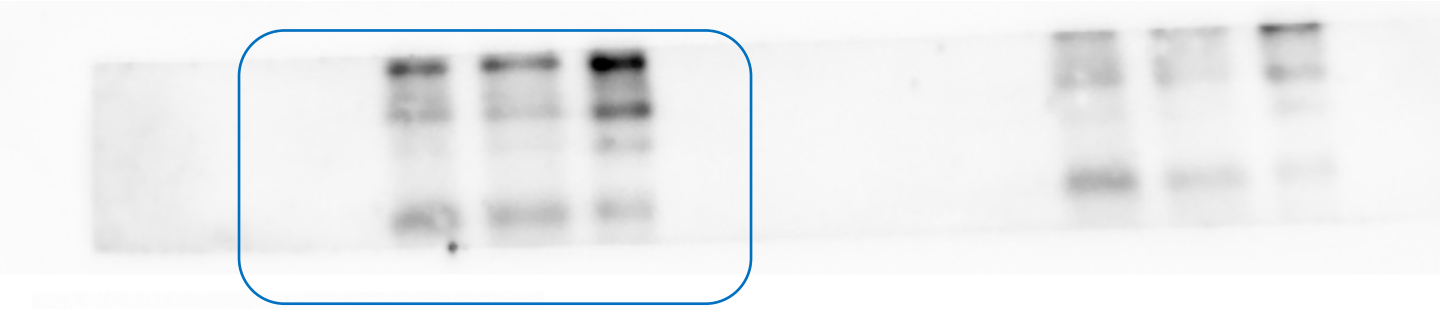

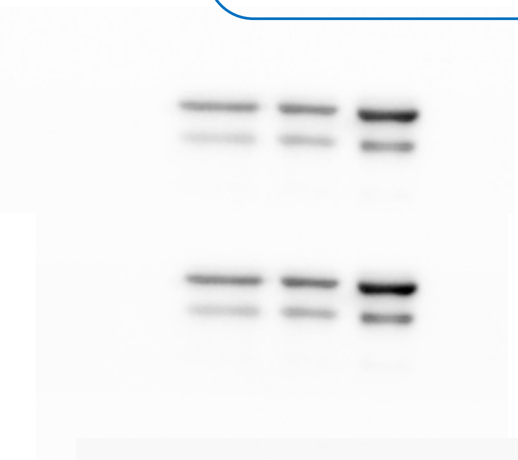

ERK （multi-exposure）

ERK （multi-exposure）

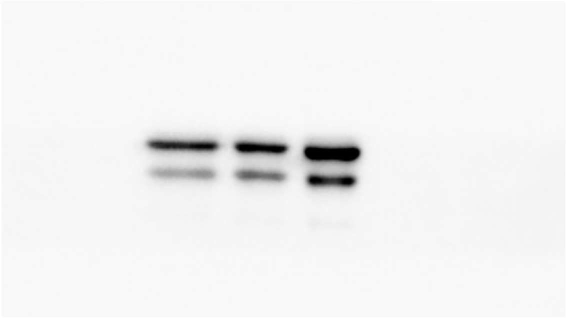

ERK （multi-exposure）

H1299 Cells

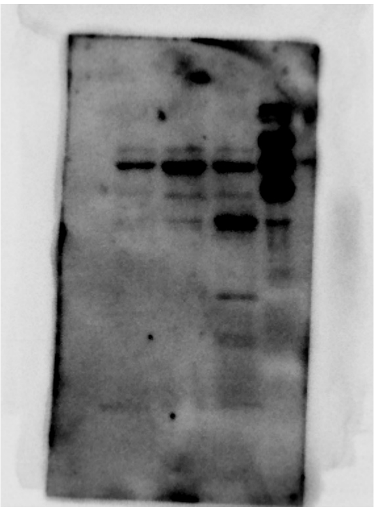

ERK

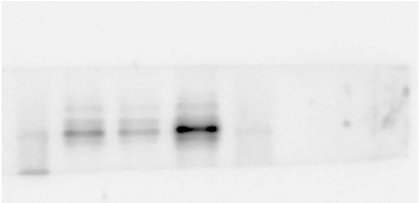

ERK

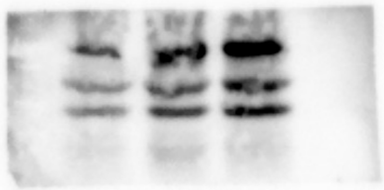

JNK

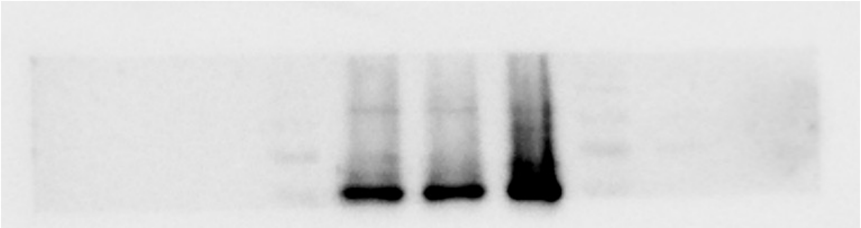

JNK

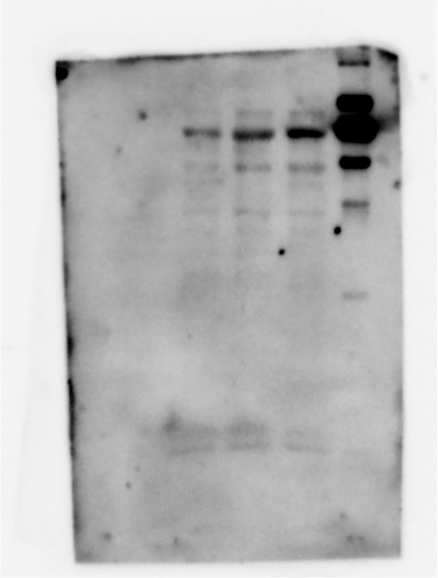

JNK

H1299 Cells

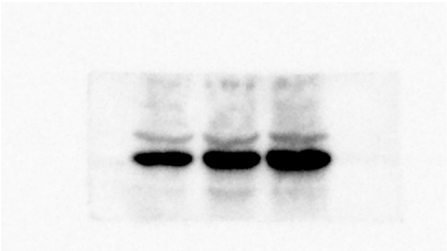

P38

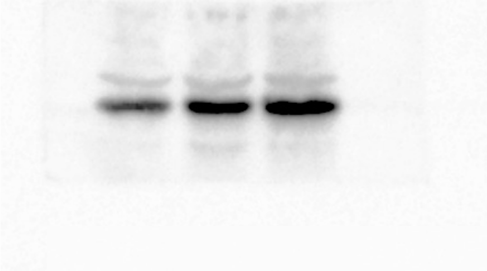

P38

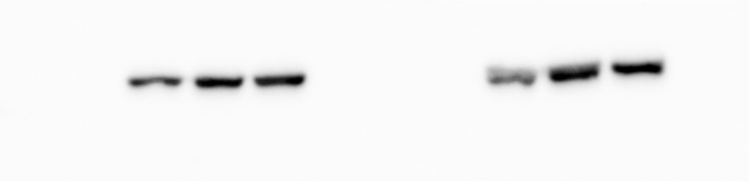

P38

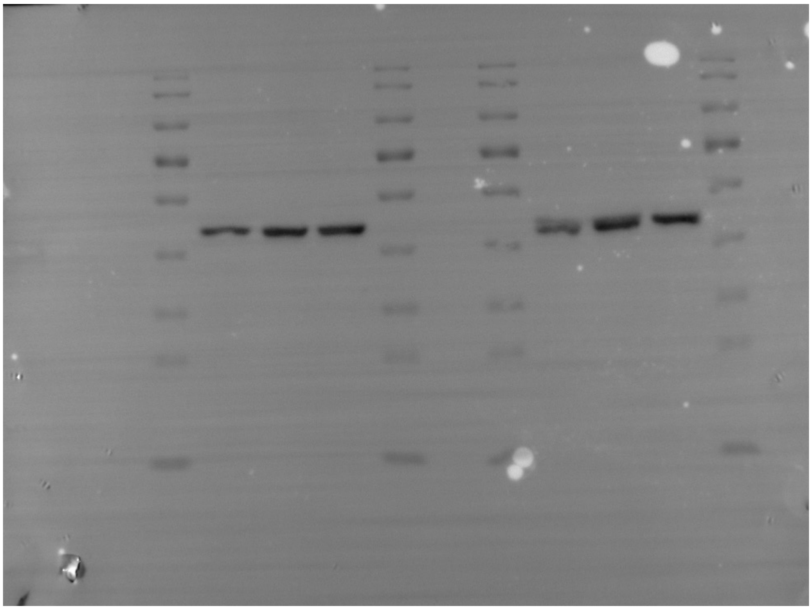

P38

H1299 Cells

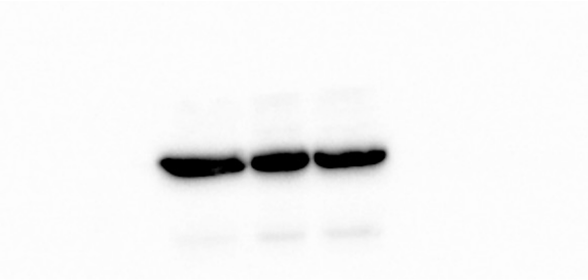

β-actin

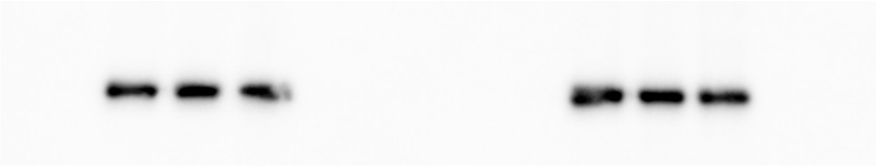

β-actin

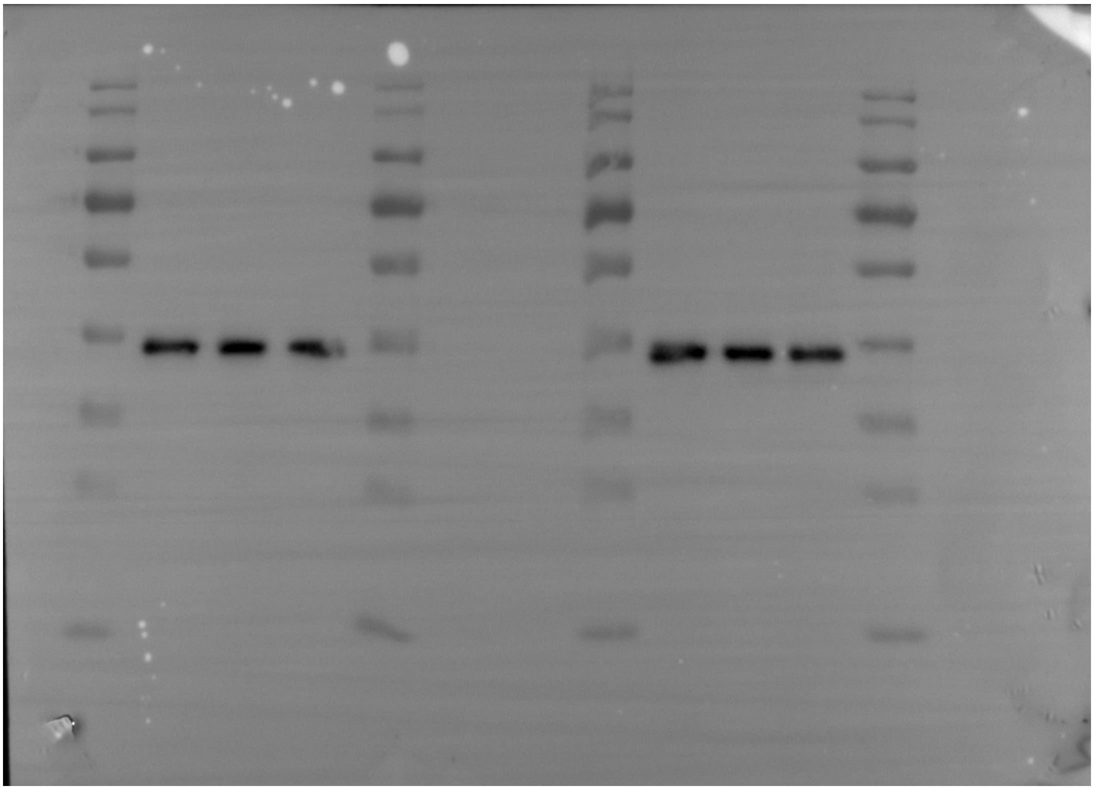

A549 Cells

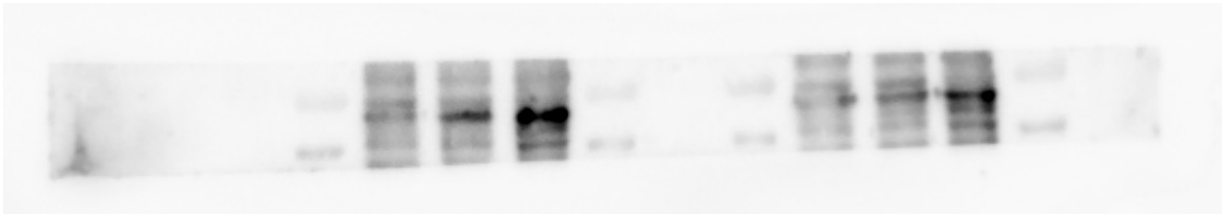

PPP3CC

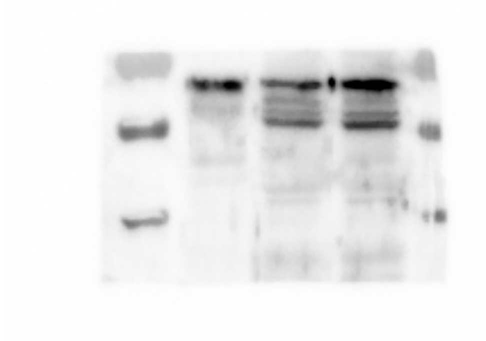

PPP3CC

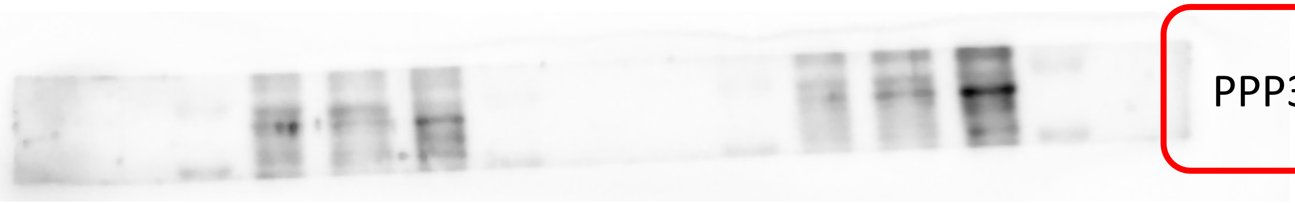

PPP3CC

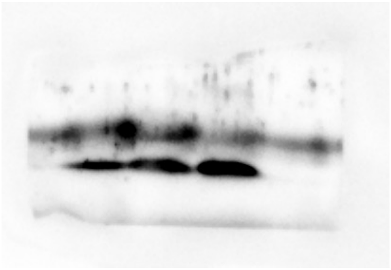

GADD45B （ multi-exposure ）

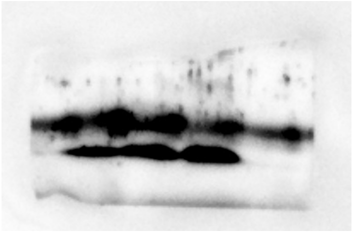

GADD45B （ multi-exposure ）

A549 Cells

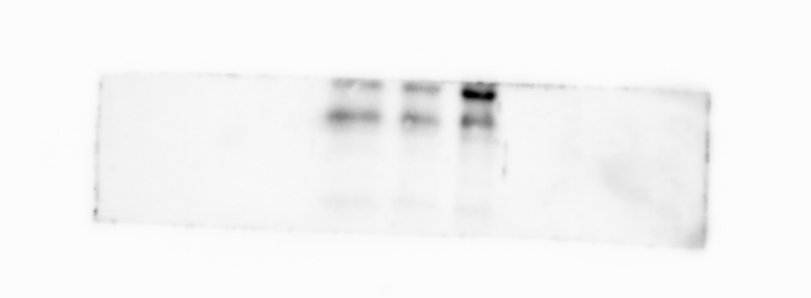

GADD45B

GADD45B

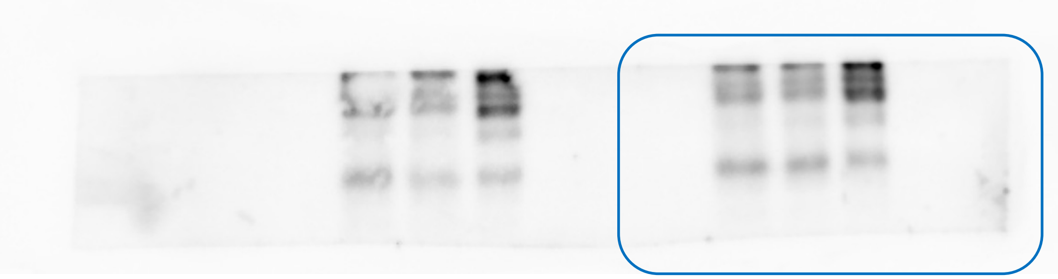

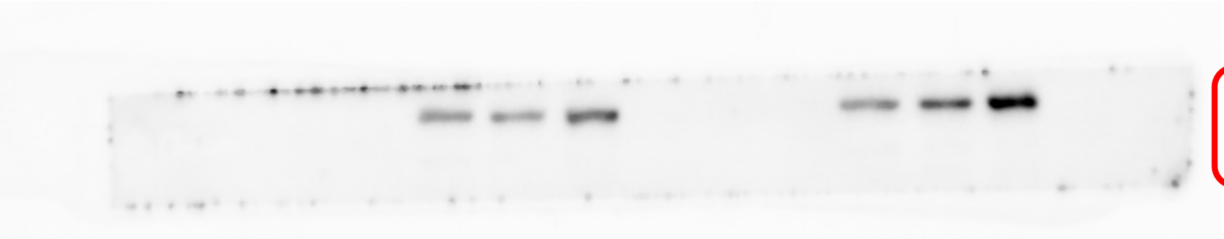

ERK

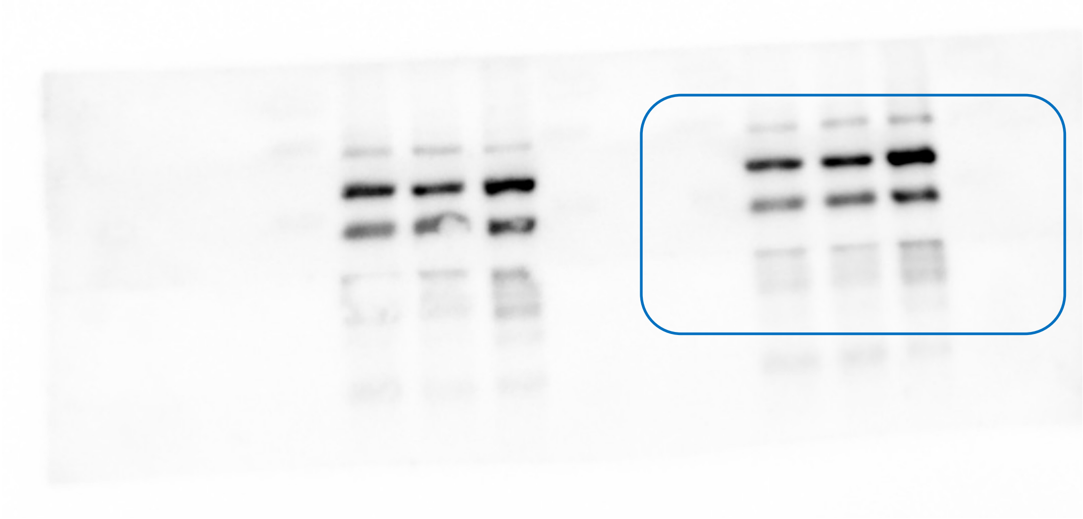

ERK

A549 Cells

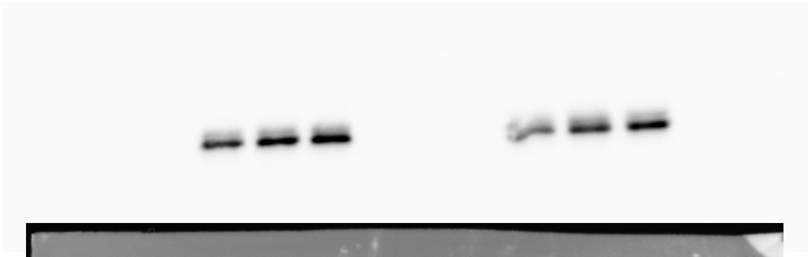

ERK

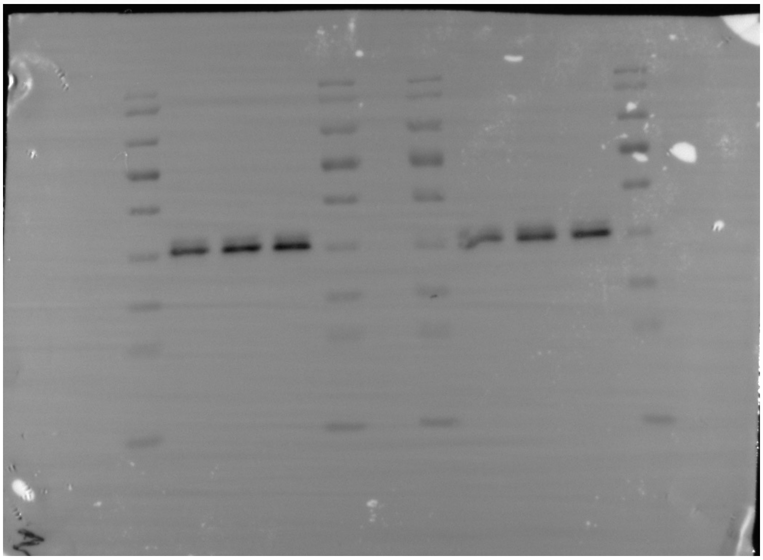

ERK

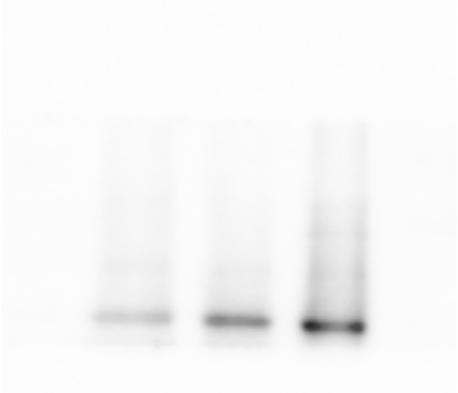

JNK （multi-exposure）

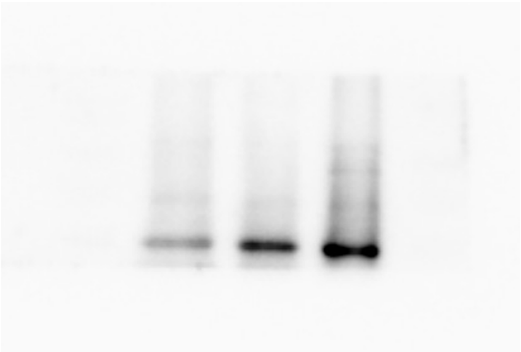

JNK （multi-exposure）

A549 Cells

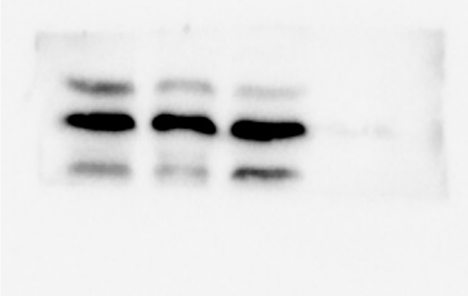

JNK

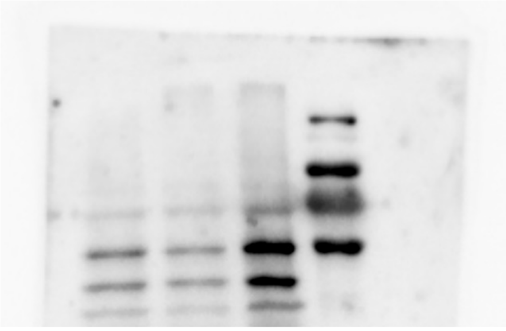

JNK

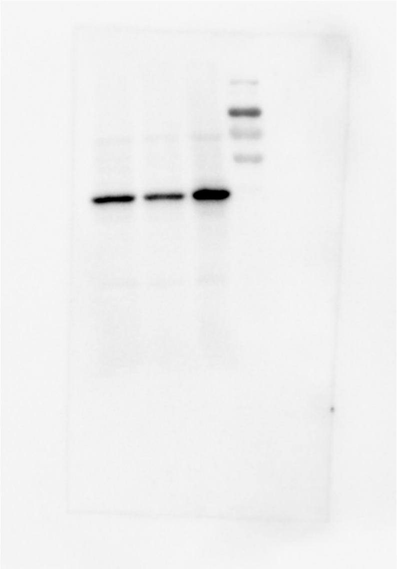

P38

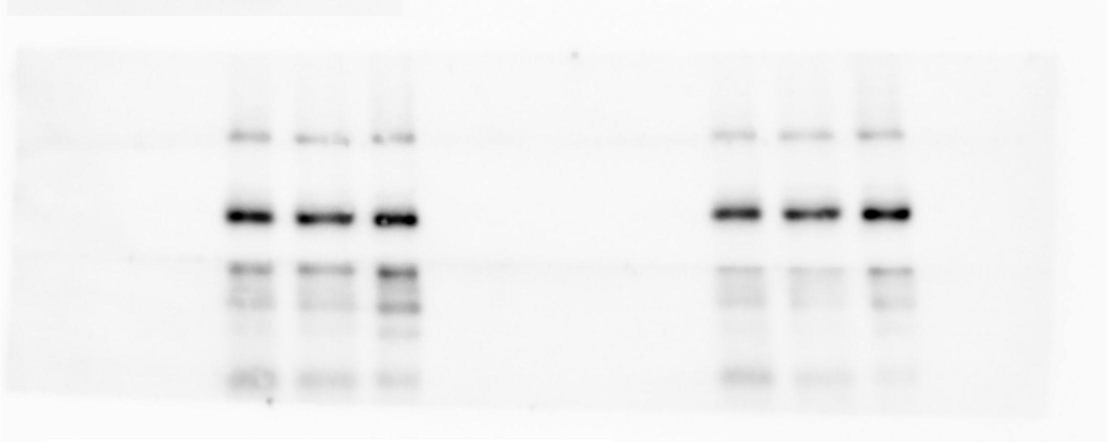

P38

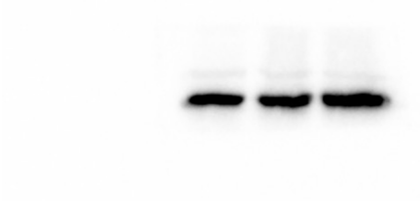

P38

A549 Cells

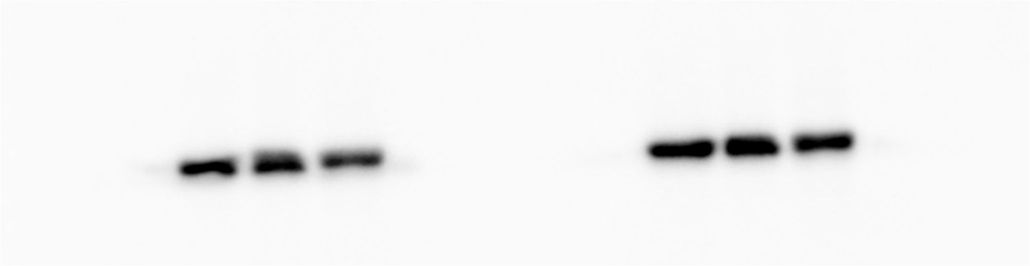

β-actin

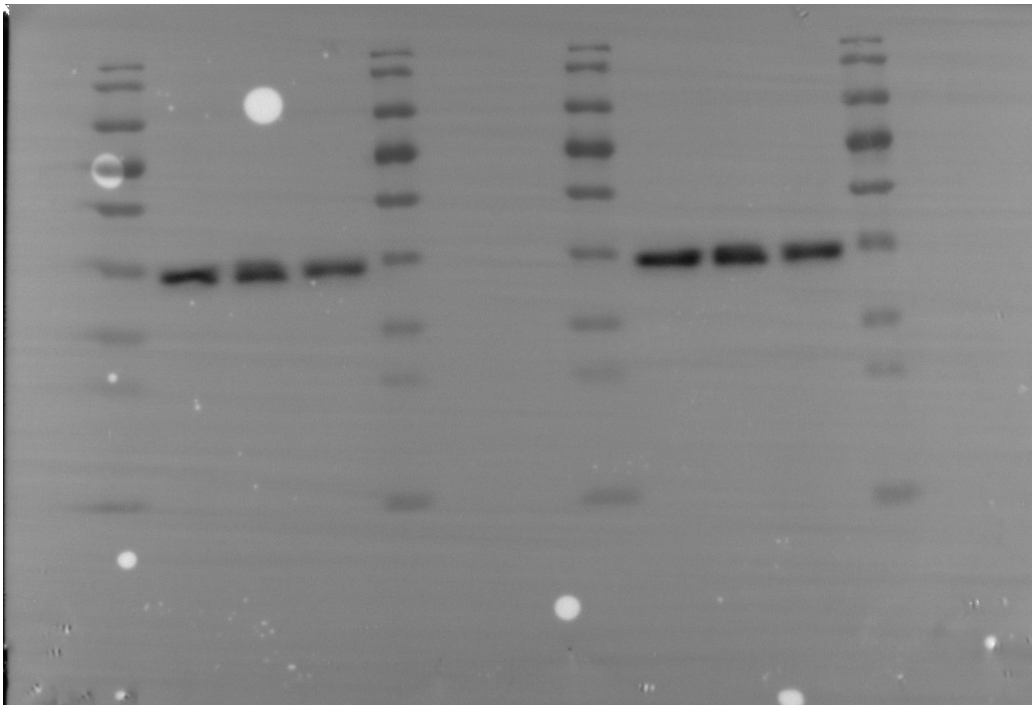

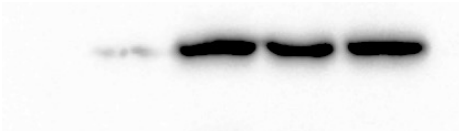

β-actin
